# Obesity-related metabolic dysfunction in dogs: a comparison with human metabolic syndrome

**DOI:** 10.1186/1746-6148-8-147

**Published:** 2012-08-28

**Authors:** Asta Tvarijonaviciute, Jose J Ceron, Shelley L Holden, Daniel J Cuthbertson, Vincent Biourge, Penelope J Morris, Alexander J German

**Affiliations:** 1 Department of Animal Medicine and Surgery, Veterinary School, Regional Campus of International Excellence "Campus Mare Nostrum", University of Murcia, 30100, Murcia, Spain; 2 Department of Obesity and Endocrinology, University of Liverpool, Leahurst Campus, Chester High Road, Neston, Wirral, CH64 7TE, United Kingdom; 3 Department of Obesity and Endocrinology, University of Liverpool, University Hospital Aintree, Longmoor Lane, Fazakerley, Liverpool, L9 7AL, United Kingdom; 4 Royal Canin Research Center, B.P.4 – 650 Avenue de la Petite Camargue, 30470, Aimargues, France; 5 The WALTHAM Centre for Pet Nutrition, Freeby Lane, Waltham-on-the-Wolds, Melton Mowbray, LE14 4RT, UK

**Keywords:** Canine, Insulin resistance, Weight loss, Adiponectin

## Abstract

**Background:**

Recently, metabolic syndrome (MS) has gained attention in human metabolic medicine given its associations with development of type 2 diabetes mellitus and cardiovascular disease. Canine obesity is associated with the development of insulin resistance, dyslipidaemia, and mild hypertension, but the authors are not aware of any existing studies examining the existence or prevalence of MS in obese dogs.

Thirty-five obese dogs were assessed before and after weight loss (median percentage loss 29%, range 10-44%). The diagnostic criteria of the International Diabetes Federation were modified in order to define canine obesity-related metabolic dysfunction (ORMD), which included a measure of adiposity (using a 9-point body condition score [BCS]), systolic blood pressure, fasting plasma cholesterol, plasma triglyceride, and fasting plasma glucose. By way of comparison, total body fat mass was measured by dual-energy X-ray absorptiometry, whilst total adiponectin, fasting insulin, and high-sensitivity C-reactive protein (hsCRP) were measured using validated assays.

**Results:**

Systolic blood pressure (*P* = 0.008), cholesterol (*P* = 0.003), triglyceride (*P* = 0.018), and fasting insulin (*P* < 0.001) all decreased after weight loss, whilst plasma total adiponectin increased (*P* = 0.001). However, hsCRP did not change with weight loss. Prior to weight loss, 7 dogs were defined as having ORMD, and there was no difference in total fat mass between these dogs and those who did not meet the criteria for ORMD. However, plasma adiponectin concentration was less (*P* = 0.031), and plasma insulin concentration was greater (*P* = 0.030) in ORMD dogs.

**Conclusions:**

In this study, approximately 20% of obese dogs suffer from ORMD, and this is characterized by hypoadiponectinaemia and hyperinsulinaemia. These studies can form the basis of further investigations to determine path genetic mechanisms and the health significance for dogs, in terms of disease associations and outcomes of weight loss.

## Background

Recently, metabolic syndrome (MS) has gained attention in human medicine given its associations with development of diabetes mellitus and cardiovascular diseases [1]. Central obesity (determined by waist circumference) is critical to its definition, in conjunction with dyslipidaemia, hypertension and glucose intolerance. This suggests that MS is a complex cluster of metabolic risk factors that together may predispose to development of secondary diseases [1]. In humans, MS doubles the risk of cardiovascular disease whilst the risk of diabetes mellitus is increased five-fold [2]. More recently, proteinuria (or microalbuminuria) has been observed in patients with MS, suggesting altered renal function [3-5].

In veterinary medicine, equine MS is well described [6-8], and known to be a risk factor for laminitis amongst other pathologies [9,10]. Canine obesity is associated with the development of insulin resistance, altered lipid profiles, and mild hypertension, which are ameliorated by weight loss [11-13]. Furthermore, overweight dogs are more likely to suffer from diabetes mellitus [14], whilst lifelong overfeeding leads to being overweight, metabolic disturbances and decreased lifespan [15]. Although laboratory dogs, rendered obese by overfeeding, have many features that resemble human MS [13], the authors are not aware of any studies examining the existence of MS in obese dogs. Therefore, the objective of the present study was to determine whether canine obesity-related metabolic dysfunction (ORMD, using a definition modified from that used for human MS) could be identified in pet dogs with naturally-occurring obesity, and whether it correlated with specific patient characteristics (e.g. signalment, body fat content), metabolic (e.g. adiponectin, insulin) and inflammatory (e.g. high-sensitivity C-reactive protein; hsCRP) biomarkers. We also determined the effect of weight loss on MS and its consequences.

## Methods

### Animals

Dogs were referred to the Royal Canin Weight Management Clinic, University of Liverpool UK, for investigation and management of obesity and associated metabolic disorders. Sixty-five dogs were recruited between February 2005 and August 2010, and those successfully losing weight had completed by January 2011. Eligibility criteria included confirmation of obesity (e.g. body fat mass >35%, as measured by dual-energy X-ray absorptiometry; DEXA; [16]), completing a weight loss programmed, and having sufficient surplus plasma to enable analyses to be completed.

Ultimately, a group of 35 dogs fulfilled the inclusion criteria. The median age was 72mo (12 to 132mo); twenty dogs were male (19 neutered) and 15 were female (13 neutered). Nine of the dogs were Labrador retrievers, and a range of other breeds were also represented including Akita, Border Collie, Cairn Terrier, CKCS (3), Cocker Spaniel, Corgi, Dachshund, Doberman (2), English Bull Terrier, Golden Retriever, Irish Setter, Lhasa Apso, Miniature Schnauzer, Mixed Breed (3), Pug (3), Samoyed, Schipperke, and Yorkshire Terrier (2). None of the dogs enrolled participated in a previous study examining metabolic effects of obesity [12], but many participated in a separate study examining renal biomarkers [17].

The study protocol adhered to the University of Liverpool Animal Ethics Guidelines, and was approved by both the University of Liverpool Research Ethics Committee and the WALTHAM ethical review committee. Owners of all participating animals gave informed written consent.

### Weight loss regimen

Full details of the weight loss regimen have been previously described [12,18]. Briefly, dogs were determined to be systemically well, and without significant abnormalities on complete blood count, serum biochemical analysis and urinalysis. Throughout weight loss, patients were weighed on electronic weigh scales (Soehnle Professional), which were regularly calibrated using test weights (Blake and Boughton Ltd). The degree of adiposity was estimated, clinically, using a 9-integer body condition score (BCS) system [19].

The weight management protocol has been previously described in detail [12,18], and involved using either a high protein high fiber (Satiety Support, Royal Canin; 34 dogs) or a high protein moderate fiber (Obesity Management, Royal Canin; 1 dog) weight loss diet (Table 1). The initial food allocation for weight loss was determined by first estimating maintenance energy requirement (MER = 440 kJ [105Kcal] × body weight [kg]^0.75^/day; [20] using the estimated target weight. The exact level of restriction for each dog was then individualized based upon gender and other factors (i.e. presence of associated diseases), and was typically between 50-60% of MER at target weight [18]. Owners also implemented lifestyle and activity alterations to assist in weight loss. Dogs were reweighed every 7-21 days and changes made to the dietary plan if necessary, until their target weight was reached [12,18].

**Table 1:** Composition of diets used for weight loss

***Criterion***	**HPHF diet**		**HPMF diet**	
ME content*	2900 Kcal/kg		3275 Kcal/kg	
*Moisture*	9.0		9.0	
	Per 100 g DM	g/1000 Kcal (ME)	Per 100 g DM	g/1000 Kcal (ME)
*Moisture*	8	28	9	27
*Crude protein*	30	103	34	104
*Crude fat*	10	34	10	31
*Crude fibre*	17.5	60	11.5	35
*Total dietary fibre*	28	97	18.5	56
*Ash*	5.3	18	7.9	24
*Fibre sources*	Cellulose, beet pulp, FOS, psyllium husk, diet cereals		Cellulose, beet pulp, diet cereals	

*HPHF* = High protein high fibre diet^d^. *HPMF* = High protein medium fibre^e^. *ME* = Metabolisable energy content, as measured by animal trials according to the American Association of American Feed Control Officials protocols; *DM* = dry matter; *FOS* = fructo-oligo-saccharides.

### Body composition analysis

Body composition was analyzed before and after the weight loss regime in all dogs, using fan-beam DEXA (Lunar Prodigy Advance; GE Lunar). Pre-weight loss total body composition results were used to estimate target weight [18,21]. Further, by comparing pre- and post-weight loss DEXA scan results, change in fat and lean mass could be estimated [12,18].

### Definition of metabolic syndrome

The guidelines of the International Diabetes Federation [22] were modified in order to produce an accessible system for dogs. Accordingly, we defined ORMD, using the either the upper limit of the reference range for the laboratory used (e.g. cholesterol, triglyceride, and glucose), or based upon internationally accepted criteria defining borderline results (e.g. SBP) [23]. Therefore, the final definition was as follows:

a) BCS 7-9/9

b) AND any two of the following:

1. Triglycerides >200 mg/dL, (2.3 mmol/L).

2. Total cholesterol > 300 mg/dL (7.8 mmol/L).

3. Systolic blood pressure >160 mmHg.

4. Fasting plasma glucose >100 mg/dL (5.6 mmol/L), or previously diagnosed type 2 diabetes mellitus.

The prevalence of metabolic syndrome before and after weight loss was assessed based upon these criteria.

### Indirect blood pressure measurement

Blood pressure was measured non-invasively using an oscillometric method (Cordell Veterinary Monitor 9401BP; Paragon Medical). All dogs were fully conscious, and either in a sitting position or in dorsal recumbence. A cuff, of appropriate size (~40% width of the leg) was used, and placed on the right forelimb. Once the dog was calm and not moving, at least five readings were taken and averaged to produce a result for systolic blood pressure (SBP).

### Clinical sampling and biochemical parameters

Blood samples were collected by jugular venepuncture prior to and after the weight loss period. All blood samples were taken after a fast of at least 16 h. Plasma cholesterol, triglycerides, and fasting plasma glucose concentrations were performed in an automated clinical chemistry analyzer (Olympus AU2700, Olympus Diagnostic GmbH) with intra and inter-assay CVs <2% for all the analyses.

Plasma adiponectin and insulin concentrations were determined using ELISA kits (Human Adiponectin ELISA, High Sensitivity Kit, BioVendor-Labaratorni Medicine for adiponectin; and Insulin, Canine ELISA, Mercodia AB, Uppsala, Sweden for insulin). Intra and inter-assay coefficient of variations (CVs) were < 11% and < 8% for adiponectin and insulin, respectively [24,25]. High sensitivity CRP (hs-CRP) was measured with a time-resolved immuno fluorometric assay with intra and inter-assay CVs of <14% [26].

### Assessment of the consequences of ORMD in dogs

In order to assess the significance of ORMD, comparisons were made between groups of dogs defined as having and not having metabolic syndrome. The parameters assessed included, signalment data, starting body weight, pre-weight loss body fat percentage, pre-weight loss plasma metabolic biomarkers (e.g. insulin, CRP, and adiponectin), rate of weight loss, energy intake required for weight loss, and change in lean tissue mass during weight loss.

### Statistical analysis

Data are expressed as median (range) except where indicated. Statistical analyses were performed with computer software (Stats Direct version 2.6.8; Stats Direct Ltd.), with the level of significance set at *P* < 0.05 for two-sided analyses. The Shapiro-Walk test was first used to assess whether or not data were normally distributed and, given that the majority of datasets were not normally distributed, non-parametric tests were chosen for all analyses. Differences in the concentrations of the various biomarkers, prior to and after weight loss, were assessed with the Wilcoxon signed rank sums test, whilst differences in the number of dogs affected by ORMD pre- and post-weight loss were compared with an exact test for matched pairs [27]. Comparisons between groups of dogs with and without ORMD were made with the Mann–Whitney test. Finally, a possible association between pre-weight loss adiponectin and insulin concentrations was tested with Kendall’s rank correlation.

## Results

### Baseline characteristics and outcomes of weight loss

Median body weight prior to weight loss was 32.9 kg (5.4 to 77.0 kg), and decreased to 25.6 kg (4.4 to 51.4 kg) after weight loss. Median BCS decreased from 8 (7-9) prior to weight loss to 5 (4 to 6) after weight loss. Weight loss took a median of 259 days (91 to 674 days), and a median weekly rate of 0.8%/week (0.2 to 1.4%). As a result, median percentage weight loss was 29% (9% to 44%). The choice of weight loss diet had no effect on the results obtained (data not shown).

### Changes in body composition and plasma metabolic biomarkers with weight loss

Median total body fat percentage decreased significantly upon weight loss (median change -53%, range -16 to -78%, P < 0.001). Although some dogs gained lean tissue mass during the process, most lost lean tissue, and the median difference was estimated to be -7% (range -21% to 9%, *P* < 0.001).

BCS (*P* < 0.001), SBP (*P* = 0.008), plasma cholesterol concentration (*P* = 0.003), plasma triglyceride concentration (*P* = 0.018), plasma insulin concentration (*P* < 0.001), and UPCR (*P* = 0.034) all decreased after weight loss (Table 2), whilst plasma adiponectin concentration increased (*P* = 0.001). Plasma hs-CRP (*P* = 0.822) and plasma glucose (*P* = 0.166) concentrations did not change with weight loss.

**Table 2:** Pre- and post-weight-loss metabolic biomarkers in the study dogs

**Criterion**	**Pre-weight loss**	**Post-weight loss**	***P***^***1***^
**BCS**^**1**^	8 (7-9)	5 (4-6)	<0.001
**SBP**^**2**^**(mmHg)**	155 (108-220)	130 (105-180)	0.008
**Cholesterol (mmol/L)**	5.6 (2.5-9.3)	5.0 (1.9-7.7)	0.003
**Triglycerides (mmol/L)**	1.2 (0.6-5.3)	0.9 (0.1-2.1)	0.018
**Glucose (mmol/L)**	5.4 (3.5-8.7)	5.2 (3.0-7.4)	0.166
**Insulin (pmol/L)**	256 (36-687)	135 (24-626)	<0.001
**Adiponectin (μg/mL)**	7.8 (0.8-19.5)	8.0 (1.1-34.9)	0.001
**hsCRP (nmol/L)**	9.1 (0.1-225.5)	9.1 (0.0-193.6)	0.822

Results are expressed as median (range). ^1^ Body condition score assessed with a 9-integer scale [19]. ^2^ Systolic blood pressure, measured indirectly by an oscillometric technique. ^3^ Urine protein:creatinine ratio. For UPCR, due to lack of availability of urine samples, was measured on 30/35 dogs. All results are expressed as mean, median (range) & number above upper limit; the exception is except for body condition score (BCS), where the mean values are not reported as the data were categorical. hsCRP: high-sensitivity C-reactive protein.

### Identification of dogs with ORMD

When different ORMD criteria were studied individually, all dogs had a BCS 7-9/9 prior to weight loss, whereas the BCS range after weight loss was 4-6/9 (Table 3). Although, after weight loss, the BCS of 6 dogs was 6/9 (i.e. above the optimal range for healthy dogs of BCS 4-5/9, [15], there was no association with persistence of abnormal metabolic parameters (data not shown). Prior to weight loss, occasional results, above respective upper reference limits, were noted for cholesterol (4/35) and triglycerides (3/35), but these were no longer evident after weight loss. In contrast, increases in SBP (10/35) and plasma glucose (11/35) were more common prior to weight loss, and many remained above the upper limit after weight loss (SBP 8/35 and glucose 7/35) (Table 3). When BCS and 4 parameters (i.e. triglycerides, cholesterol, SBP, and glucose) were used in the definition of ORMD, 7/35 fulfilled the criteria before weight loss (Table 3).

**Table 3:** The number of animals with BCS, SBP, cholesterol, triglyceride, and glucose above the upper reference limit pre- and post-weight-loss

**Criterion**	**Upper limit**^**1**^	**Pre-weight loss**	**Post-weight loss**
**Body condition score**^**2**^	>6/9	35	0
**Cholesterol**	>7.8 mmol/L	4	0
**Triglycerides**	>0.23 mmol/L	3	0
**SBP**^**3**^	>160 mmHg	10	8
**Glucose**	>5.5 mmol/L	11	8

^1^ Upper limit: the upper reference range limit used for the definition of metabolic syndrome. ^2^ Body condition score assessed with a 9-integer scale [19]. ^3^ Systolic blood pressure, measured indirectly by an oscillometric technique. All results are expressed as mean, median (range) & number above upper limit; the exception is except for body condition score (BCS), where the mean values are not reported as the data were categorical.

### Comparison of obese dogs with and without ORMD pre-weight loss

To determine factors associated with ORMD baseline data, pre-weight loss body composition analysis results, weight loss outcomes and plasma metabolic biomarker concentrations were compared between dogs that either did or did not fit the definition. Plasma adiponectin concentration was less in dogs with metabolic syndrome (*P* = 0.031), whilst plasma insulin concentration was greater (*P* = 0.030; Table 4), and they were negatively correlated with one another (Kendall’s tau -0.29, *P* = 0.016). However, none of the other parameters, including assessment of total and regional body fat or weight loss rate differed between groups (*P* > 0.1 for all).

**Table 4:** Comparison pre-weight loss parameters in obese dogs with and without metabolic syndrome

**Criterion**	**Metabolic syndrome**	**No metabolic syndrome**	***P***
**Age mo**	98 (31 to 132)	66 (12 to 132)	0.335
**Sex**^**1**^	5 NM, 2 NF	1 M, 14 NM, 2 F, 11 NF	0.835
**Breed**^**2**^	Dachshund, Doberman, English Bull Terrier, Irish Setter, Labrador, Lhasa Apso, Mixed Breed	Akita, Border Collie, Cairn Terrier, CKCS (3), Cocker Spaniel, Corgi, Doberman, Golden Retriever, Labrador (8), Miniature Schnauzer, Mixed Breed (2), Pug (3), Samoyed, Schipperke, Yorkshire Terrier (2)	0.321
**Start weight kg**	42.8 (8.5 to 56.3)	28.6 (5.4 to 77.0)	0.479
**Total BF**^**3**^**%**	47 (37 to 51)	44 (30 to 55)	0.424
**Weight loss %**	28 (16 to 44)	28 (9 to 38)	0.531
**Weight loss rate %/wk**	0.7 (0.2 to 1.4)	0.8 (0.3 to 1.4)	0.642
**EI during weight loss**^**4**^	62 (53 to 74)	60 (44 to 74)	0.672
**Change in lean mass %**	-7 (-21 to -2)	-7 (-20 to 10)	0.537
**Adiponectin μg/mL**	4.1 (2.4 to 8.1)	8.4 (0.8 to 19.5)	0.031
**Insulin pmol/L**	428 (125 to 686)	222 (36 to 524)	
**μIU/mL**	62 (18 to 99)	32 (5 to 75)	0.030
**hs-CRP nmoL/L**	7.0 (0.1 to 56.6)	11.1 (4.0 to 214.8)	0.719

^1^ For sex, *F*: female, *NF*: neutered female, *NM*: neutered male. Upper limit: the upper reference range limit used for the definition of metabolic syndrome. ^2^ For Breed, *CKCS*: Cavalier King Charles spaniel. ^3^ Total body fat measured by dual-energy X-ray absorptiometry. ^4^ Energy intake for weight loss is the median value for the whole of weight loss, expressed as energy (in Kcal) per kg metabolic body weight (kg^0.75^)/day. All results are expressed as median (range).

## Discussion

Human MS is now well recognized and predisposes to cardiovascular diseases and type 2 diabetes mellitus [1]. In equine MS, obesity, regional adiposity, insulin resistance, hypertriglyceridaemia and hyperleptinaemia are part of the definition, and it is a risk factor for laminitis, altered reproductive function, and seasonal alterations in arterial blood pressure [6,9]. In the current study, we classified pet dogs with naturally-occurring obesity on the basis of presence of ORMD (using a modification of criteria used for human metabolic syndrome). We then examined the effect of weight loss on these metabolic criteria, and sought to identify factors that were potentially associated with the syndrome. The criteria for ORMD were met in approximately 20% of obese patients pre-weight loss. The presence of ORMD was not associated with total fat mass, as measured by DEXA, but was associated with increased and decreased plasma insulin and adiponectin concentrations, respectively. These findings suggest that defining obese dogs on the basis of their metabolic status may have some merit, although further work is now required to determine the true significance of ORMD in terms of disease risk and outcome. Weight loss was associated with decreased body fat mass, BCS, SBP, circulating lipid concentrations, and plasma insulin concentration, whilst circulating adiponectin increased. These findings are similar to those reported in many studies of canine obesity [11-13,28], and suggest that the study population was representative. Nonetheless, the study was small and extending the work further studies would now be desirable.

We chose to base our ORMD definition on the guidelines of the International Diabetes Federation [22], although other guidelines are available including those of the World Health Organization [29], the European Group for the Study of Insulin Resistance [30], the National Cholesterol Education Program [31], and a combined statement from the American Heart Association and National Heart, Lung, and Blood Institute [32]. The main reason for this was that the criteria used could readily be adapted to produce a practical method in dogs. For each criterion used, and in a similar manner to humans, either the upper limit of the respective laboratory reference ranges (e.g. cholesterol, triglyceride, and glucose), or internationally accepted criteria, above which the parameter is borderline (e.g. SBP) [23] was chosen as the cut-off. Whilst many of the criteria were identical to those used in the human system, others were substituted for similar parameters thought to be more relevant for dogs. The main advantages of such an approach were that all chosen parameters are already in 2widespread clinical use and techniques for measurement are better validated, thereby making the whole system more accessible for practicing veterinarians. For example, we replaced waist circumference (a human measure of central obesity), with BCS, since the significance of central obesity has not been studied in dogs. Given differences in canine anatomy, the human measures of central adiposity are not likely to be appropriate for dogs, and developing a clinical measure of abdominal obesity in dogs (e.g. waist circumference) would be challenging given the wide variability in size and shape amongst breeds. In contrast, differences in BCS are associated with both disease risk and decreased longevity [14,15]. Nonetheless, it may be necessary to modify these criteria in the future, if a practical method of measuring central obesity can be validated.

In a similar manner, total plasma cholesterol concentration was used in place of HDL-cholesterol. The main reason for this modification was the fact that lipid profiles differ between dogs and humans, with humans demonstrating an ‘LDL pattern’, whilst HDL is the dominant cholesterol type in dogs [33]. Further, alterations in lipoprotein profiles differ in human and canine obesity: obese humans display increased LDL-cholesterol and decreased HDL-cholesterol [1], whereas the circulating concentrations of both LDL and HDL-cholesterol are increased in obese dogs [11,34]. Thus, the use of total cholesterol, rather than cholesterol fractions, is more logical for the latter species.

Based upon definitions of 4 (i.e. SBP, cholesterol, triglyceride and glucose) parameters in addition to BCS, approximately 20% of the obese dogs of the study were classified with ORMD, respectively. This suggests that the prevalence of MS in obese dogs is somewhat less than for humans where prevalence is typically 22-28% and 50-60% in overweight and obese patients, respectively [35]. The reasons for such a difference are not known, and further elucidation of the underlying mechanisms of ORMD is recommended for comparative purposes.

In order to determine the significance of ORMD, we chose to assess a variety of other parameters including fasting insulin concentration, plasma adiponectin concentration, CRP and adiposity, as determined by DEXA. When the obese dogs of the present study were subdivided on presence or absence of ORMD prior to weight loss, only plasma adiponectin and insulin concentrations differed. Adiponectin was approximately twofold less, and insulin approximately twofold greater, in the ORMD group, and both were negatively correlated with one another. This finding is similar to that described in the human literature on MS [36]. In man, one of the major obesity and MS outcomes is insulin resistance and type 2 diabetes mellitus [1], and the risk of this condition is increased fivefold when MS is present [2]. The exact pathogenetic link between obesity and insulin resistance has not yet been fully elucidated [37]. However, adiponectin is known to have an insulin-sensitizing effect, acting through the AMP-activated protein kinase [38], so that the association with MS may (at least in part) be explained by the decreased adiponectin concentration that accompanies obesity. Adiponectin may also have anti-inflammatory effects, such that decreased adiponectin concentrations are associated with the increased risk of inflammation [39]. Thus, hypoadiponectinaemia observed in human MS may be responsible for development of secondary diseases due to increased susceptibility to inflammation and insulin resistance. Interestingly, no difference was noted in hsCRP, either when comparisons were made before and after weight loss, or when obese dogs were categorized as either having or nor having concurrent ORMD. This may suggest that associations amongst adiponectin, MS and obesity are independent of the function of this particular acute phase protein. Further, these findings are different from some previous studies examining CRP concentrations in obese dogs, where increased [12,40] or decreased [13] concentrations have been seen. The reasons for such differences are not entirely clear, but may have resulted from differences in the test populations and assay used. Most notably, our work utilized a high-sensitivity assay recently validated for dogs [26], and such assays are thought to be more reliable in humans [41]. The findings regarding adiponectin concentrations in the current study are different from some [12,42] but not other [43] studies. Again, the reasons for this are not clear but similar explanations would be feasible, namely that this related to population differences or assay type. In the present study, we used a high sensitivity human adiponectin ELISA assay previously validated for use in dogs [34]. In this assay, human calibrators were substituted for species-specific standards, in order to achieve similar affinity of antiserum against standards; this ensures that better differentiation between samples with greater and lesser adiponectin concentrations [34]. Nonetheless, high and low molecular weight adiponectin species were not measured in the current study, and these may differ in importance is development of obesity-associated consequences [44]. Thus, the true significance of these findings requires further study.

The presence of increased plasma insulin concentration, and decreased plasma adiponectin concentration implies physiological consequences to ORMD. However, in order to determine its true significance, further investigations would be needed examining other biomarkers and also clinical consequences. For example human MS is associated with dysregulated fatty acid metabolism [45], cardiac and vascular functional derangements [46], and hepatic manifestations such as non-alcoholic fatty liver disease [47]. Therefore, future studies could assess alterations in a variety of biomarkers in a prospective population of dogs. Epidemiological studies could also be considered, as a means of determining potential disease associations and, ultimately, risk of death in ORMD.

Another interesting observation from the current study was the fact that no differences in fat mass were identified between dogs classified with or without MS. This implies that canine obesity does not inevitably lead to metabolic dysfunction, and is similar to findings in man, where some obese individuals are determined to be metabolically healthy [48]. These patients are not insulin resistant, are normotensive, and have normal plasma concentrations of triglyceride, glucose, high-sensitivity C-reactive protein (hsCRP), and high-density lipoprotein (HDL) cholesterol. The reasons why some dogs may be protected from developing metabolic derangements of obesity are not known and require further study. Possible explanations, not examined in the current study include the age of onset obesity, time taken to become obese, duration of obesity prior to weight loss, and the type of diet fed during the obese stage. As a result, further work is required to determine the mechanisms involved in the development of ORMD.

As is often the case, this study has limitations that should be considered. The main study limitation was that client-owner out bred dogs were used, which undoubtedly added to study variability. For instance, environment, diet, exercise and husbandry were variable. Most notable is the issue of diet, since the amount of food eaten, and type of diet could have influenced the pre-weight-loss metabolic parameters, particularly cholesterol and triglycerides. Unfortunately, the information obtained from the owner regarding diet fed before enrolment was vague and incomplete. A range of foods was fed, including commercial pet food, treats, and human food. Further, owners frequently fed many different diets, rarely measured the amount fed out accurately, and did not record the extra food fed. As a result, it was not possible to generate a meaningful record of pre-weight-loss feeding that could be used in this study. Further, information was unclear as to the exact duration of obesity in many cases and, again, this may have influenced the results obtained. Moreover, ethical limitations meant that we were unable to perform more invasive but gold standard assessments of insulin sensitivity such as hyperinsulinaemic, euglycaemic clamps or minimal model analysis to determine insulin sensitivity [49,50]. That said, the obesity had developed naturally and been longstanding (e.g. >12 months) in most cases, which is arguably more representative of the at-risk population of interest. Nonetheless, it would be sensible to consider further studies as a means of elucidating the underlying mechanisms of ORMD more precisely.

## Conclusions

In conclusion, this study has described that up to a third of obese dogs do suffer from ORMD, which is characterized by hypoadiponectinaemia and hyperinsulinaemia. This study can form the basis of further investigations to determine pathogenetic mechanisms and the health significance for dogs, in terms of disease associations and outcomes of weight loss.

## Competing interests

The following conflicts of interest apply: AJG’s Senior Lectureship is funded by Royal Canin; the diet used in this study is manufactured by Royal Canin; PJM is an employee of WALTHAM, whilst VB is employed by Royal Canin.

## Authors’ contributions

AT – performed laboratory assays, drafted manuscript, and reviewed the manuscript; JJC – performed laboratory assays, drafted the manuscript, and reviewed the manuscript; SLH – collected clinical data, and reviewed manuscript; DJC – Assisted with results interpretation, and reviewed the manuscript; PJM – designed the study, reviewed the results, and reviewed the manuscript; VB – designed the study, reviewed the results, and reviewed the manuscript; AJG – designed the study, collected the clinical data, analyzed the results, and drafted the manuscript. All authors have approved the final article.
